# Autologous Platelet-Rich Plasma for Endometrial and Ovarian Dysfunction: Mechanistic Insights and Clinical Outcomes

**DOI:** 10.3390/biomedicines14102323

**Published:** 2026-10-13

**Authors:** Xiaofang Qin, Ting Yang, Lu Bai, Yuanyuan Xin, Xiaohui Liu, Haofei Shen, Yanjiao Yao

**Affiliations:** 1 Department of Gynaecology and Obstetrics, The First Hospital of Lanzhou University, Lanzhou University, Lanzhou 730000, China; qinxf970805@163.com (X.Q.);; 2 Department of Geriatrics, National Clinical Research Center for Geriatrics, West China Hospital, Sichuan University, Chengdu 610041, China; 3 Department of General Practice, Cheling Town Central Health Center, Mingshan District, Ya’an 625106, China

**Keywords:** platelet-rich plasma, thin endometrium, diminished ovarian reserve, angiogenesis, assisted reproductive technology

## Abstract

Although assisted reproductive technology (ART) has been widely applied worldwide, thin endometrium (TE), diminished ovarian reserve (DOR), and other conditions remain the core bottlenecks of refractory infertility, and existing therapies fail to effectively preserve tissue and prevent functional damage. Platelet-rich plasma (PRP), as an autologous regenerative agent, has demonstrated promising application potential in reproductive medicine due to its multi-targeted regulatory capacity. Therefore, this review is intended as a narrative overview that aims to systematically elucidate the possible mechanisms by which PRP improves the endometrial microenvironment and ovarian function, integrate the latest clinical evidence to evaluate its therapeutic efficacy, analyze translational challenges such as the lack of standardization, and provide an evidence-based basis for its standardized clinical application.

## 1. Introduction

In recent years, assisted reproductive technology (ART) has achieved technological iteration and clinical popularization globally, providing core fertility solutions for infertile families. However, thin endometrium (TE), recurrent implantation failure (RIF), diminished ovarian reserve (DOR), and intrauterine adhesion (IUA) remain the core clinical bottlenecks that urgently need to be broken through in the field of reproductive medicine. They are also the key factors in ART cycle cancellation, embryo implantation failures, and pregnancy losses. Epidemiological data show that the incidence of TE (midluteal endometrial thickness ≤ 7 mm) is approximately 2–3% in IVF cycles [1], and insufficient endometrial thickness can lead to adverse perinatal outcomes such as reduced pregnancy rate and live birth rate [2]. RIF, defined as a clinical phenomenon characterized by a lack of implantation after the transfer of several embryos, affects approximately 10% of the ART population, and there is currently no standard diagnostic and treatment protocol [3]. The incidence of DOR is increasing with the delay in female childbearing age and iatrogenic injuries, and due to its unclear mechanism and complex clinical features, there is still a lack of effective means to reverse follicular depletion [4], which significantly restricts pregnancy outcomes. In addition, chronic endometritis (CE), premature ovarian insufficiency (POI), and severe oligoasthenoteratozoospermia in males further complicate the treatment of infertility. The common pathological basis of these challenges lies in the damage to local tissue microvascular networks, decreased stem cell activity, and imbalance of extracellular matrix remodeling, which urgently require novel intervention strategies with multi-targeted regenerative potential.

In the 1990s, Marx et al. first proposed the concept of platelet-rich plasma (PRP) in the field of regenerative medicine, aiming to explore the therapeutic potential of platelet-derived growth factors in tissue healing [5]. With its characteristics of being enriched by centrifugation of autologous peripheral blood, low immunogenicity, and high concentrations of growth factors and cytokines, it has been successfully translated into clinical practice in orthopedics, dermatology, and wound regeneration [6]. In the past five years, the regenerative potential of PRP has been innovatively introduced into the field of reproductive medicine. After activation, it releases active molecules such as VEGF, PDGF, TGF-β, IGF-1, and IL-10, which can synergistically regulate angiogenesis, establish an anti-inflammatory microenvironment, and recruit tissue stem cells through paracrine pathways [7]. Multiple clinical observational studies and early randomized controlled trials (RCTs) have preliminarily provided evidence suggesting that intrauterine PRP infusion can significantly improve endometrial thickness and effectively enhance pregnancy outcomes in patients with thin endometrium [8]. Local ovarian injection of PRP has shown the potential to increase antral follicle count (AFC) and the number of retrieved oocytes in some patients with DOR [9]. Local application may also be associated with exploratory value in improving semen parameters, cryopreservation effects, reducing oxidative stress, and improving the testicular microenvironment in males [10].

Despite the promising regenerative potential of PRP in reproductive medicine, its clinical translation is still limited by insufficient standardization of preparation and weak mechanistic evidence. Currently, there are significant differences among studies in terms of blood collection volume, centrifugation gradient, leukocyte retention strategy (P-PRP/L-PRP), and activator selection, leading to high heterogeneity in platelet concentration multiples and growth factor release profiles [11], which severely impairs the comparability between studies. Furthermore, the molecular mechanisms underlying PRP-mediated reproductive regulation remain incompletely elucidated, particularly the action targets, signaling networks, and time-dependent characteristics in human ovarian tissues, which warrant further investigation via multi-omics and single-cell sequencing technologies. Additionally, the absence of standardized guidelines for clinical operational parameters (including indications, administration routes, dosages, intervention timings, and treatment cycles) severely restricts its precise clinical application and safe dissemination.

Based on this, this article reviews the latest research progress of PRP therapy in reproductive medicine, elaborates on its molecular mechanisms and cellular biological effects in endometrial receptivity reconstruction and ovarian microenvironment improvement, integrates and analyzes the efficacy data of high-quality clinical studies from this year, and discusses the influencing factors, limitations, and core controversies of current PRP clinical application. This study aims to provide a theoretical reference for deepening basic research on PRP in reproductive medicine, offer an evidence-based basis for its standardized and precise clinical application, and ultimately promote the effective and safe translation of PRP technology in reproductive medicine, providing a novel therapeutic option for patients with refractory infertility. Given the broad scope and heterogeneous nature of the available evidence, this article is designed as a narrative rather than a systematic review.

## 2. Overview and Preparation Technology of PRP

### 2.1. Definition and Classification System

Platelet-rich plasma refers to a plasma derivative obtained by centrifugation of autologous peripheral venous blood with a platelet concentration significantly higher than the baseline level (usually 2–7 times) [12]. According to leukocyte content and fibrin network structure, PRP and its derivative products can be systematically divided into four categories: pure platelet-rich plasma (P-PRP), leukocyte- and platelet-rich plasma (L-PRP), pure platelet-rich fibrin (P-PRF), and leukocyte and platelet-rich fibrin (L-PRF) [13]. The core biological activity of PRP originates from the abundant bioactive molecules stored in platelet α-granules, mainly including transforming growth factor beta (TGF-β1), platelet-derived growth factor (PDGF), vascular endothelial growth factor (VEGF), and insulin-like growth factor (IGF), which can synergistically drive tissue regeneration, angiogenesis, immune regulation, and extracellular matrix remodeling [14]. These biological characteristics make PRP a promising regenerative intervention in reproductive medicine, which can specifically address the core pathological bases of refractory infertility caused by endometrial and ovarian lesions, including damage to microvascular networks, decreased stem cell activity, and imbalance of extracellular matrix remodeling.

### 2.2. Standardized Preparation: A Critical Appraisal of Current Heterogeneity

Although the two-step gradient centrifugation method is the mainstream approach for PRP preparation in reproductive medicine [15], a critical examination of the literature reveals that there is no universally accepted “gold standard” [16]. A scoping review reported marked heterogeneity across all steps of PRP preparation, encompassing anticoagulant selection, centrifugation protocols, activation methods, and other critical parameters [17]. This heterogeneity severely compromises the comparability of clinical studies and impedes the establishment of evidence-based guidelines.

The variability in PRP preparation encompasses multiple interrelated domains.

1. Variations in centrifugation parameters (relative centrifugal force, duration, temperature) can lead to differences in the platelet concentration and growth factor content of the product. When comparing the first centrifugation parameters (RCF 200–400 g, temperature 5–19 °C, duration 5–19 min) to the second centrifugation parameters (RCF 200–800 g, duration 5–19 min, temperature fixed at 12 °C), Amable et al. found that these different parameters resulted in a fluctuation of platelet recovery rates ranging from 46.9% to 69.5%, with the final platelet concentration varying from 1.4 × 10^6^ to 1.9 × 10^6^ platelets/μL (an increase of 5.4 to 7.3 times) [18]. Importantly, a systematic review found that centrifugation RCF was reported in only 6.7% of included studies, highlighting a critical reporting deficit [6].
2. Activation methods also vary considerably (calcium alone, calcium/thrombin, or thermal activation), with significant effects on cytokine release kinetics. Calcium/thrombin activation increases overall cytokine release over seven days, while calcium-only activation sustains VEGF and IGF concentrations in double-spin preparations [19].
3. Although there has been ongoing debate over the pros and cons of including white blood cells in PRP [20,21], studies by Kobayashi et al. have found that the level of growth factors is significantly influenced by the concentration of white blood cells: leukocyte concentration positively correlates with PDGF-BB and VEGF but negatively with FGF-β, and MMP-9 (a catabolic factor) correlates only with leukocyte concentration, not platelet concentration [22]. This finding underscores the need for method selection according to target pathology.
4. Anticoagulant choice (EDTA, ACD-A, sodium citrate) and commercial preparation systems further contribute to product heterogeneity. Citrate-based anticoagulants are preferred over EDTA (which may damage platelet membranes) [23], and a comparison of seven commercial systems found that platelet and leukocyte concentrations differed largely between systems, with large differences observed for all growth factors both between and within systems [24]. European regulations, governed by directives such as 2002/98/EC and 2005/62/EC, require adherence to Good Manufacturing Practices for blood and blood components, including PRP [25]. Failure to strictly adhere to manufacturing specifications may result in suboptimal platelet concentrations within PRP, thereby affecting its therapeutic efficacy.

In summary, variability in centrifugation parameters, activation methods, leukocyte content, anticoagulants, and commercial systems yields PRP products with divergent biological compositions across studies. This heterogeneity represents a major barrier to evidence-based translation of PRP therapy in reproductive medicine. To ensure safety, efficacy, and reproducibility, unified quality control and reporting standards are urgently needed.

## 3. Improving the Microenvironment of the Endometrium Through PRP

### 3.1. Mechanism of Action (Figure 1)

The core of PRP improving the endometrial microenvironment lies in the release of bioactive substances such as growth factors, anti-inflammatory cytokines, and matrix metalloproteinases after enrichment by autologous blood centrifugation and in vitro activation, completing structural repair and functional remodeling of the damaged endometrium through multi-targeted and multi-pathway synergistic cascade effects [26]. High levels of VEGF and PDGF-BB in PRP bind to VEGFR2 and PDGFRβ receptors on endothelial cells, activating the PI3K/Akt and ERK1/2 signaling pathways, which significantly enhances endometrial spiral artery branching and capillary network density, thereby reversing insufficient blood perfusion in the thin endometrium [27,28]. While promoting angiogenesis, PRP releases abundant growth factors, including VEGF, PDGF and TGF-β, and upregulates the expression of multiple proliferation markers in endometrial stromal cells, namely cytokeratin, Ki-67 and HOXA10 [29], stimulating endothelial cell proliferation and inducing endometrial regeneration. Building on cellular proliferation and angiogenesis, PRP releases chemokines, including CCL5, CCL7, CXCL13 and lipoxin A4, and simultaneously inhibits the phosphorylation of NF-κB [30,31]. This effectively suppresses excessive inflammatory responses associated with chronic endometritis, establishes the immune-tolerant microenvironment required for embryo implantation, and prevents fetal rejection. The establishment of immune homeostasis further regulates the dynamic homeostasis of the ECM: the expression levels of COL1A1, TIMP-1, TGF-β1 and TNF-α are significantly downregulated, while the expression of MMP1, MMP3, MMP7 and MMP26 is upregulated [28]. By modulating the expression ratio of these factors, PRP promotes functional collagen deposition while inhibiting pathological fibrosis, providing structural support for tissue integrity reconstruction after hysteroscopic adhesiolysis. Additional studies have demonstrated that PRP can also inactivate the TGF-β1-SMAD2/3 signaling pathway and downregulate the expression of fibrosis-related genes [32]. It should be noted that the mechanistic insights presented in this section are derived from a combination of preclinical studies (including animal models and in vitro experiments using human-derived cell lines) and clinical observational data. Unless otherwise specified, findings related to specific signaling pathways (e.g., PI3K/Akt, TGF-β1-SMAD2/3) are primarily based on preclinical evidence, whereas endometrial receptivity markers (e.g., HOXA10, LIF) and clinical outcomes have been validated in human studies.

**Figure 1:**
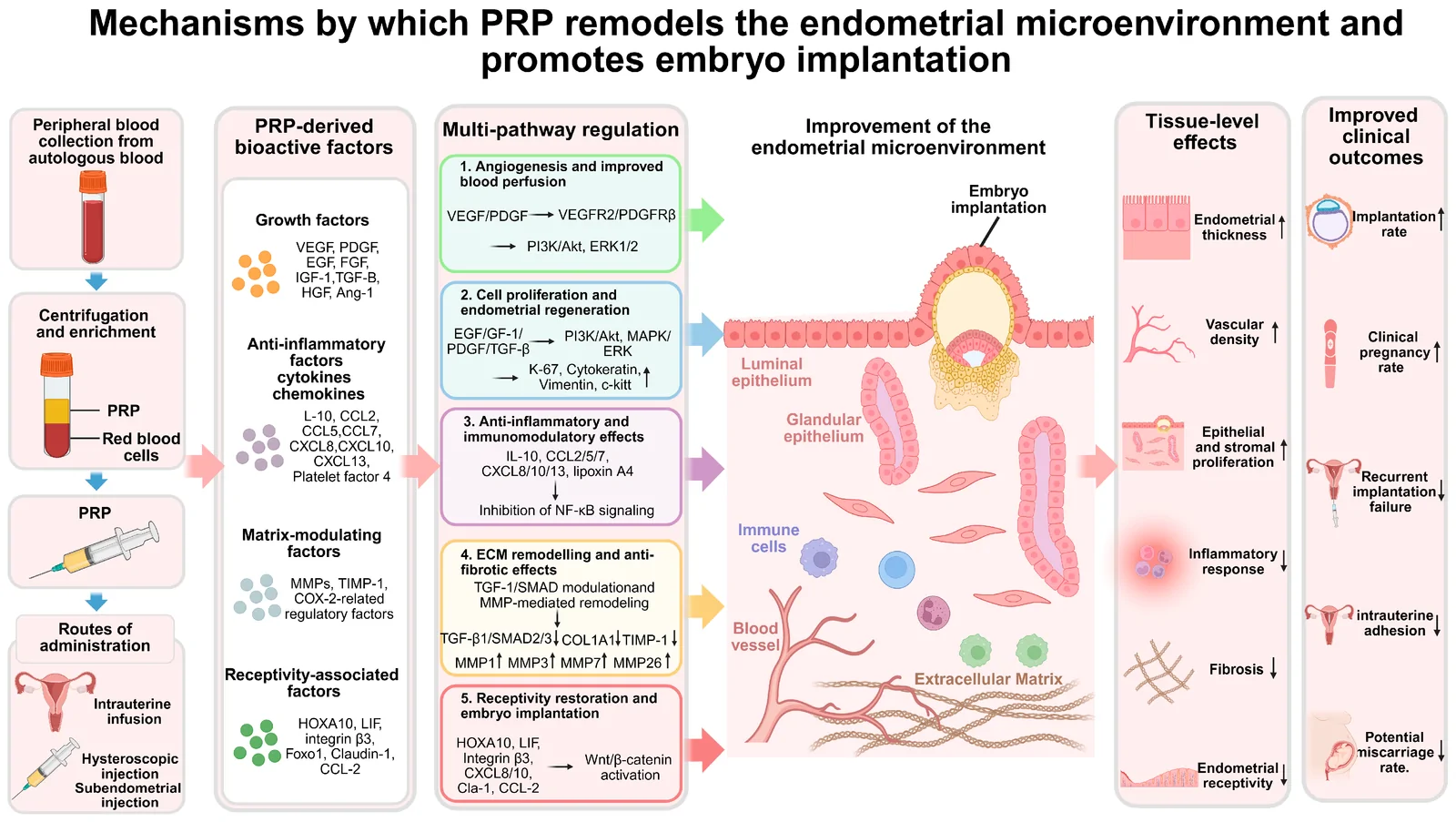
Mechanism by which PRP remodels the endometrial microenvironment and promotes embryo implantation. This figure depicts the preparation of PRP from autologous peripheral blood through collection and centrifugation: (1) angiogenesis and improved blood perfusion; (2) cell proliferation and endometrial regeneration; (3) anti-inflammatory and immunomodulatory effects; (4) ECM remodelling and antifibrotic effects; (5) endometrial receptivity restoration. These regulatory actions ultimately produce tissue-level improvements: increased endometrial thickness, elevated vascular density, enhanced epithelial and stromal proliferation, attenuated inflammatory response, reduced fibrosis and improved endometrial receptivity. Consequently, PRP treatment significantly elevates embryo implantation rate and clinical pregnancy rate, while decreasing the risks of recurrent implantation failure, intrauterine adhesion and potential miscarriage.

Ultimately, A10. LIF and integrin β3 during the window of implantation, but also releases chemokines including CCL2, CXCL8, CXCL10 and claudin-1 [33], optimizing the embryo-endometrium molecular compatibility and thereby establishing a stable pregnancy microenvironment for RIF patients.

### 3.2. Clinical Efficacy (Table 1)

Intrauterine application of PRP has become an important regenerative treatment for endometrial-derived infertility, such as TE, RIF, and IUA. Its core clinical benefits are concentrated in the significant increase in endometrial thickness and the substantial improvement in clinical pregnancy rate. Multiple recently published high-quality randomized controlled trials (RCTs) and meta-analyses have provided data supporting its clinical application value in assisted reproductive technology [33,34].

**Table 1:** The preceding data in the table are from the control group (patients received routine basic care without PRP treatment), while the subsequent data are from the experimental group (patients received PRP intrauterine perfusion or subendometrial injection). Both groups include subjects’ basic information (age, infertility duration), core uterine assessment indicators (endometrial thickness, endometrial pattern, blood flow resistance index before and after treatment), and pregnancy outcome data (embryo implantation rate, clinical pregnancy rate, miscarriage rate), providing direct evidence for quantifying and comparing the clinical efficacy of PRP in improving endometrial receptivity and enhancing pregnancy success rate.

Clinical Intervention		Age (Years, *p* > 0.05)		BMI (kg/m^2^, *p* > 0.05)		Duration of Infertility (Years, *p* > 0.05)		Endometrial Thickness (mm, *p* < 0.05)		Clinical Pregnancy Rate (%) (*p* < 0.05)		Source DOI
1.0 mL on day 11	1.0 mL on both days 11 and 13	30.7	29.6	22.96	22.37	3.91	4.47	7.96	8.42	27	48.9	[35]
HRT treatment	Hysteroscopic injection of autologous PRP.	39.0	40.0	21.80	22.41	1.6	3	6.7	8.7	22	52	[36]
Before PRP treatment	Post-PRP treatment	own control	38.6	own control	22.1	own control	4.21	5.0	6.1	3.3	31.9	[37]
Before PRP treatment	Post-PRP treatment	own control	37.07	own control	22.44	own control	1.64	6.7	7.6	20.2	37.1	[38]
Before PRP treatment	Post-PRP treatment	own control	38.4 ± 4.3	own control	23.3	own control	5.7	5.4	6.0 (*p* = 0.070)	0	30	[39]
Standard HRT	Standard HRT in conjunction with intrauterine infusion of PRP gel	36	37	22.1	21.6	3	2	6.3	6.7	18.5	38.3	[40]
Infusible PRP Group	Injectable PRP Group	39.16	37.26	26.2	24.5 (*p* = 0.0321)	5.2	3.4	7.41	8.31	0	40	[41]
Did not receive PRP	PRP therapy	/	/	/	/	/	/	/	The endometrial thickness in the RG was higher than that in the CG (*p* < 0.05).	20	44.12	[42]
Estradiol valerate	Intrauterine instillation of autologous PRP	29.11	29.2	20.9	21.1	4.17	4.28	5.89	7.47	/	/	[43]
Before PRP treatment	Post-PRP treatment	own control	38.6	own control	22.3	own control	2.8	6.3	7.9	/	/	[44]
The 10th day of the second HRT cycle after PRP administration	The 14th day of the second HRT cycle after PRP administration	own control	39.7	own control	21.19	/	/	5.85	7.25	/	15.6 (after PRP)	[45]
Before PRP treatment	Post-PRP treatment	own control	36.25	/	/	/	/	3.97	6.63	/	/	[46]
Before PRP treatment	PRP injections	own control	35.1	/	/	/	/	5.3	7.4	/	/	[27]
Did not receive PRP	Hysteroscopic injection of autologous PRP	32.7	35	25.9	25.3	6.5	4.5	/	/	13.33	20	[47]
Standard treatment	PRP treatment	33.61	34.11	25.19	24.73	4.6	4.3	/	/	19.28	48.97	[48]
1 mL of G-CSF	1 mL autologous PRP	32.7	31.6	26.24	26.12	6.1	4.9	/	/	13	33.7	[49]
Did not receive PRP	PRP treatment	33.19	33.49	21.69	21.99	3	4	/	/	47.44	65.85	[50]
Did not receive PRP	LP-PRP intrauterine infusion	32.22	32.04	22.52	20.69	/	/	/	/	24.5	45.3	[51]

(1) Improvement effect of PRP on endometrial thickness

TE with endometrial thickness ≤ 7 mm in the mid-luteal phase is an important clinical indicator leading to ART cycle cancellation and embryo implantation failure [27]. Its core pathological feature is impaired endometrial proliferation capacity and insufficient blood perfusion, and PRP can effectively stimulate endometrial growth and achieve an increase in endometrial thickness by releasing a variety of growth factors and bioactive substances [52]. A systematic review and meta-analysis including 2152 patients with thin endometrium showed that among six intervention measures compared with the control group, intrauterine PRP infusion was associated with a statistically significant positive effect in improving endometrial thickness (MD = 0.989, 95% CrI = [0.612, 1.36]), and the PRP group showed the most favorable outcomes in terms of both endometrial thickness improvement and clinical pregnancy rate increase [53]. A retrospective cohort study further suggested that a single PRP infusion was sufficient to increase the median endometrial thickness from 6.5 (IQR 1.0) to 7.3 mm (IQR 1.3) (*p* < 0.0001) overall [38].

Subgroup analysis of different administration routes showed that subendometrial PRP injection was associated with better pregnancy outcomes. Clinical data showed that the average endometrial thickness with this administration method was 8.31 mm, significantly higher than 7.42 mm in the intrauterine PRP infusion group [41]. The superior efficacy of subendometrial injection is biologically plausible, as direct delivery of platelets to the endometrial stem/progenitor cell niche at the basalis-functional interface may enhance growth factor bioavailability and tissue penetration compared to passive diffusion from the uterine cavity. In addition, dosing frequency represents an additional variable that may influence PRP efficacy. Feng et al. [35] conducted a prospective randomized trial comparing single versus double intrauterine PRP infusions in thin endometrium patients, demonstrating that the double-infusion regimen (1.0 mL on days 11 and 13 of the HRT cycle) yielded a significantly greater increase in endometrial thickness compared to a single infusion on day 11 alone (an additional 0.46 mm, *p* < 0.01), along with improved uterine hemodynamic parameters. This suggests a cumulative dose–response effect for endometrial applications.

Regarding timing, the interval between PRP administration and embryo transfer varies considerably across studies: Liu et al. administered PRP 24–72 h before FET [50], while Nazari et al. used a precise 48-h interval [48]. Given that activated PRP releases the majority of its growth factors within the first 24 h, with sustained release over 7 days [19], this 24- to 72-h variability may significantly impact the synchronization between peak growth factor concentrations and the endometrial receptivity window. However, no study has systematically compared different timing protocols, representing a critical knowledge gap.

For women with intrauterine adhesions (IUA), intrauterine PRP infusion can effectively inhibit endometrial fibrosis and promote epithelial regeneration. RCT data suggested that the increase in endometrial thickness in patients using PRP was significantly higher than that in patients not using PRP (*p* = 0.03 and 0.04) [54], laying the necessary structural foundation for embryo adhesion and invasion.

(2) Improvement effect of PRP on clinical pregnancy rate

The improvement in endometrial thickness and upregulation of receptivity ultimately translate into significant benefits in ART cycle pregnancy outcomes. A meta-analysis of 861 patients with thin endometrium, implantation, or pregnancy failure undergoing assisted reproductive technology showed that the clinical pregnancy rate in the PRP group was 2.51 times higher than that in the control group (RR: 2.51; 95% CI: 2.0–3.13; *p* < 0.00001), with no significant heterogeneity among studies (I^2^ = 0.0%, *p* < 0.00001). Moreover, the live birth rate in the PRP group was 7.03 times that in the control group (RR: 7.03; 95% CI: 3.91–12.6; *p* < 0.00001), suggesting a potential benefit of this therapeutic approach.

Data from a single-center self-controlled before-and-after study showed that the clinical pregnancy rate of patients before LP-PRP treatment was only 24.5%, which significantly increased to 45.3% after treatment (*p* = 0.022). Meanwhile, the embryo live birth rate per cycle was significantly higher than that in the control group (42.2% vs. 18.5%, *p* = 0.009), indicating clear clinical translation value [51]. It is worth noting that this study did not compare the efficacy of LP-PRP and LR-PRP at the same time, and many studies lacked a direct comparison of different PRP types in terms of reproductive indications, so it was unable to draw a clear conclusion on the optimal composition of white blood cells.

For patients with RIF, an RCT [55] including 40 subjects found that the clinical pregnancy rate in the intrauterine PRP infusion group reached 55.0%, significantly higher than 20.0% in the control group (*p* ≤ 0.05). At the same time, the intrauterine adhesion score decreased compared with that before PRP treatment, and no serious treatment-related adverse events were observed.

A systematic review and meta-analysis [33] published in 2024 further indicated that for patients receiving ART treatment, intrauterine PRP infusion not only significantly increased endometrial thickness but also was associated with the clinical pregnancy rate by 2.04 times (RR: 2.04, 95% CI: 1.52 to 2.76, *p* = 0.000). Moreover, the cycle cancellation rate in the PRP group was significantly lower than that in the control group (RR: 0.46, 95% CI: 0.23 to 0.93, *p* = 0.000), suggesting enhanced clinical pregnancy benefits.

## 4. Improving Ovarian Function Through PRP

### 4.1. Mechanism of Action (Figure 2)

PRP-mediated ovarian function restoration follows a cascade regulatory network of “microenvironmental remodeling → cell survival → metabolic reprogramming → follicle activation” [56]. Studies have indicated that PRP treatment upregulates HIF-1α expression, thereby promoting VEGF release from activated platelet α-granules, and VEGF binds to VEGFR2 on endothelial cells, which undergoes autophosphorylation to activate the VEGFA/VEGFR2/ERK pathway and trigger the PI3K/AKT and Ras/MAPK pathways, rapidly reconstructing the ovarian cortical microvascular network and alleviating tissue hypoxia [57,58,59]. At the granulosa cell level, IGF-1, PDGF and EGF synergistically activate the PI3K/Akt/mTOR survival pathway, upregulate Bcl-2 expression and significantly promote FOXO3a transcription [60,61,62], thereby facilitating follicle activation, granulosa cell proliferation, and inhibiting cellular apoptosis. Targeting the core pathology of oocyte senescence, the active components of PRP induce the upregulation of antioxidant enzyme systems, including *Cox7a2* and *Mtch2*, and downregulate the expression of Prdx2 and Numa1 proteins to scavenge mitochondrial ROS, which also activates PGC-1α to drive mtDNA replication and transcription, thereby maintaining ATP synthesis and meiotic spindle integrity [63,64].

**Figure 2:**
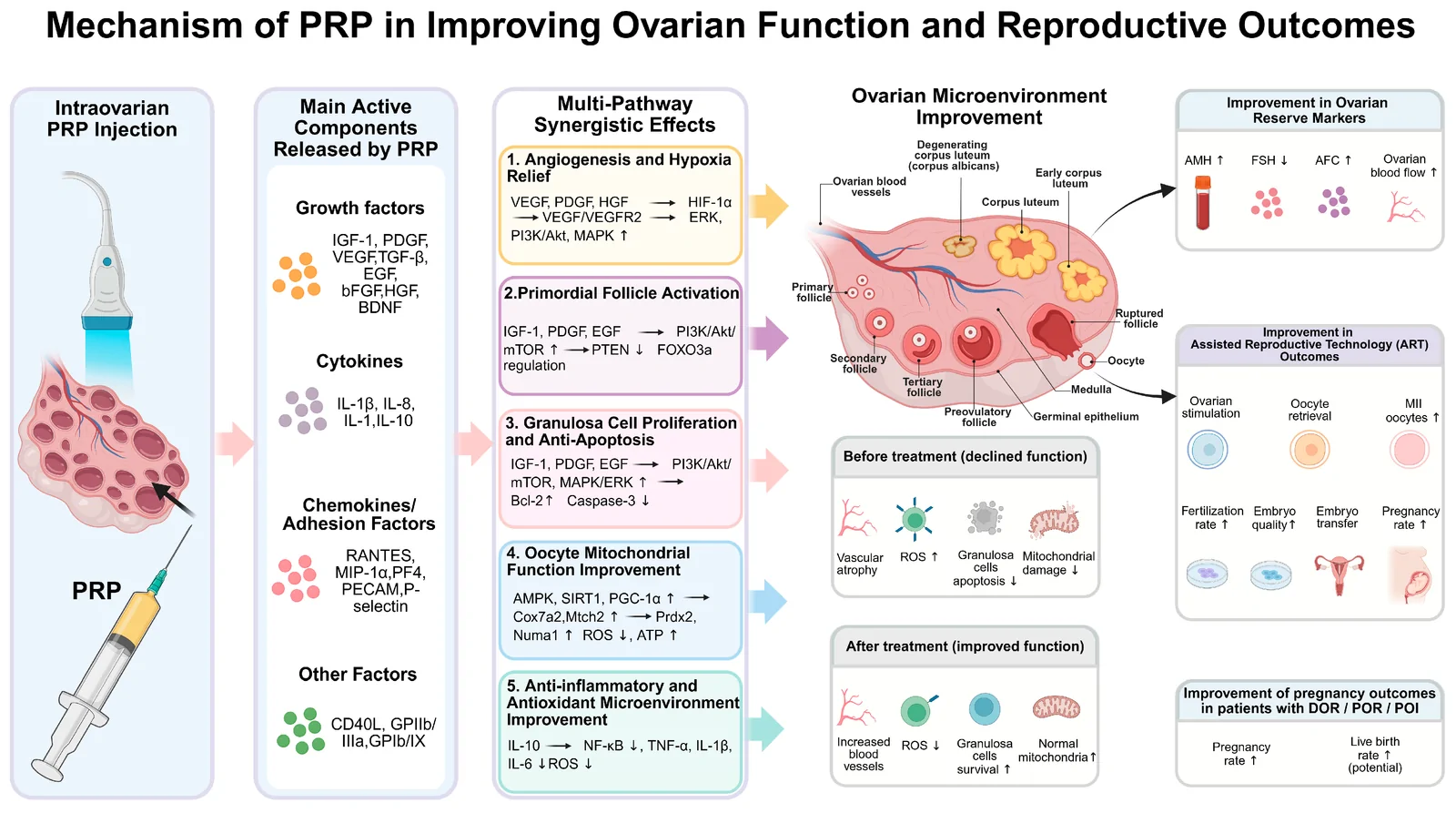
Mechanism of PRP in improving ovarian function and reproductive outcomes. This figure demonstrates that following intraovarian injection, the active components released by PRP—including growth factors, cytokines, chemokines/adhesion factors, and other bioactive molecules—exert synergistic effects via five core pathways: (1) angiogenesis and hypoxia relief; (2) primordial follicle activation; (3) granulosa cell proliferation and anti-apoptosis; (4) oocyte mitochondrial function improvement; (5) anti-inflammatory and antioxidant microenvironment optimization. These regulatory actions reverse pathological conditions such as ovarian vascular atrophy, reactive oxygen species (ROS) accumulation, granulosa cell apoptosis, and mitochondrial damage, thereby comprehensively improving the ovarian microenvironment. Ultimately, PRP treatment leads to improvements in ovarian reserve markers (anti-Müllerian hormone [AMH], follicle-stimulating hormone [FSH], antral follicle count [AFC], and ovarian blood flow), enhanced efficiency at each stage of assisted reproductive technology (ART), and significantly elevated pregnancy rates and potential live birth rates in patients with diminished ovarian reserve (DOR), poor ovarian response (POR), and premature ovarian insufficiency (POI).

These metabolic reprogramming effects ultimately converge on the precise activation of the primordial follicle pool: PRP reversibly inhibits PTEN to relieve excessive silencing of the PI3K/AKT pathway, promoting dormant follicle entry into the growth phase, which is clinically manifested as a synchronous increase in AMH and AFC [56]. Furthermore, PRP treatment significantly reduces TNF-α levels, and PRP-released cytokines further inhibit the NF-κB pathway [65], collectively establishing a hypoinflammatory, highly vascularized ovarian regenerative microecology. It should also be noted that the following mechanistic framework is primarily derived from preclinical studies, including rodent models of premature ovarian insufficiency and in vitro experiments using human ovarian tissue or granulosa cells. Where human clinical data support these mechanisms—such as changes in AMH, FSH, and AFC following intraovarian PRP injection—these are clinical correlations rather than direct mechanistic evidence.

### 4.2. Clinical Efficacy (Table 2)

Intraovarian injection of PRP is a promising regenerative intervention for refractory ovarian-derived infertility, such as DOR, poor ovarian response (POR), and POI in the field of assisted reproductive technology, significantly improving the core ovarian reserve via elevated AMH, reduced FSH and increased AFC.

**Table 2:** The preceding data in the table are from the control group, and the subsequent data are from the experimental group. Both groups include the basic information of subjects (age, infertility duration), core ovarian reserve indicators (anti-Müllerian hormone [AMH], follicle-stimulating hormone [FSH], antral follicle count [AFC] before and after treatment), and assisted reproductive technology (ART)-related outcome data, providing direct evidence for quantifying and comparing the clinical efficacy of PRP in improving ovarian function.

Clinical Intervention		Age (Years, *p* > 0.05)		BMI (kg/m^2^, *p* > 0.05)		Duration of Infertility (Years, *p* > 0.05)		AMH (ng/mL, *p* < 0.05)		FSH (*p* < 0.05)		AFC (*n*, *p* < 0.05)		Source DOI
Did not receive PRP	PRP treatment	41	41	25	24.6	/	/	0.58	1.01	15.0 mIU/L	9.07 mIU/L	5	7	[66]
Before PRP treatment	Post-PRP treatment	own control	40.3	/	/	own control	4.6	0.35	0.53	20.6 mIU/L	16.4 mIU/L	2.6	4.2	[67]
Before PRP treatment	Post-PRP treatment	own control	34.8	/	/	own control	5.6	0.13	0.18	/	/	0.5	1.7	[68]
Before PRP treatment	Post-PRP treatment	own control	36.5	own control	26.2	own control	3.8	/	/	12.9 IU/L	11.1 IU/L	0.7	3.1	[69]
Patients’ left ovaries	PRP treatment	own control	40.40	own control	22.59	own control	2.79	/	/	/	/	1.60	2.50	[70]

(1) Improvement effect of PRP on core endocrine markers of ovarian reserve

Serum AMH and FSH are the core endocrine markers for evaluating ovarian reserve function, which can objectively reflect ovarian function status from two dimensions: follicle pool reserve level and ovarian endocrine feedback regulation ability [71]. Intraovarian injection of PRP can simultaneously achieve an increase in serum AMH level and a decrease in FSH level in patients with diminished ovarian function by activating primordial follicles, improving granulosa cell function, and reconstructing the ovarian cortical microenvironment [72], providing direct endocrinological evidence for the improvement of ovarian reserve function.

A systematic review and meta-analysis [9] including 2256 patients with diminished ovarian reserve showed that after intraovarian PRP injection, the level of AMH increased (3 months MD 0.36, *p* = 0.002, 95% CI: [0.20; 0.52]) and the level of FSH decreased significantly (3 months MD −8.87, *p* = 0.010, 95% CI: [−14.19; −3.55]). In this patient population, the reported rates were 18% for clinical pregnancy and 11% for live birth.

A retrospective study of 60 patients with poor ovarian response undergoing ART showed that after ultrasound-guided intraovarian PRP injection, the baseline mean AMH level of patients significantly increased from 0.8 ng/mL to 2.7 ng/mL, the baseline mean FSH level decreased from 12.9 IU/mL to 11.1 IU/mL (*p* = 0.002), and endometrial thickness also significantly increased (from 4.4 mm to 9.6 mm) [69].

A prospective controlled, non-randomized comparative study [66] published in 2020 showed that women treated with PRP experienced a significant improvement in FSH, AMH, and AFC, whereas there was no change in the control group. Furthermore, overall rates of biochemical (26.1% versus 5.4%, *p* = 0.02) and clinical pregnancy (23.9% versus 5.4%, *p* = 0.03) were higher in the PRP group, suggesting additional benefits beyond conventional treatment.

(2) Improvement effect of PRP on antral follicle count and key clinical indicators of ART cycles

AFC is an imaging indicator that intuitively reflects the number of recruitable follicles in the ovary, is directly related to the number of retrieved oocytes and embryo quality in ART cycles, and is a core indicator for predicting ovarian responsiveness [73]. Multiple clinical studies have suggested that intraovarian PRP injection can significantly increase the AFC level in patients with diminished ovarian function: an RCT including 83 subjects showed that the average AFC of patients increased by 3.0 after PRP treatment (*p* < 0.0001) [74]. An experimental study published in 2022 further suggested that PRP injection significantly increased the mean AFC of patients (from 1.60 ± 0. 84 to 2.50 ± 0.90, *p* = 0.043) [70].

On this basis, the improvement of ovarian reserve function by PRP can be further transformed into comprehensive benefits of key clinical indicators in ART cycles: data from a prospective study showed that after PRP treatment, the number of antral follicles in patients increased from 2.6 to 4.2 (*p* < 0.001), the number of mature oocytes increased from 1.7 to 2.7 (*p* < 0.001), while the average number of IVF attempts per patient decreased from 2.8 to 1.6 (*p* < 0.001), the fertilization rate significantly increased from 57.6% to 66.9%, and the blastocyst formation rate also showed a clinically significant increase, which may improve the probability of patients entering the embryo transfer stage [67].

Similarly, the ovarian response to PRP also exhibits dose-related variability. Guo and Kawamura, in a comprehensive review of ovarian function restoration using PRP, noted that while 2 mL per ovary is a commonly used dose in clinical practice, a dose of ≥4 mL per ovary may be associated with better follicle recruitment effects in patients with severe DOR, whereas excessive volume may cause high-pressure tissue damage [75]. This suggests a potential therapeutic window rather than a simple linear dose–response relationship for ovarian PRP.

## 5. Discussion

As an autologous regenerative agent, PRP has emerged as a research hotspot in reproductive medicine for refractory endometrial lesions and diminished ovarian function, owing to its multi-targeted tissue repair and microenvironment modulation capacities. While numerous high-quality studies have provided evidence supporting its efficacy in improving endometrial receptivity, enhancing ovarian reserve, and optimizing ART outcomes, substantial research heterogeneity, incomplete mechanistic elucidation, and the absence of standardized systems still impede its clinical translation.

### 5.1. Synergy and Specificity of PRP Mechanisms in Endometrial and Ovarian Repair

This review demonstrates that the core value of PRP in reproductive medicine lies in its multi-targeted regenerative potential, which can simultaneously address the two major problems of poor endometrial receptivity and diminished ovarian reserve function. Although the target organs of action are different, there is significant synergy in their core molecular mechanisms. The aforementioned mechanisms demonstrate that PRP exerts its effects in both the endometrium and ovarian tissue by releasing high concentrations of growth factors (e.g., VEGF, PDGF, IGF-1), which drive angiogenesis and inhibit apoptosis, thereby reversing tissue hypoxia and degeneration [28]. However, there are specific differences in the primary focus of its actions between these two tissues. In the endometrial microenvironment, the primary role of PRP is to create a “favorable niche” for embryo implantation by upregulating receptivity markers (e.g., HOXA10, LIF) and modulating the immune microenvironment via inhibition of the NF-κB pathway. In contrast, in ovarian function restoration, PRP predominantly focuses on achieving metabolic reprogramming from a “quiescent” to a “proliferative” state by scavenging mitochondrial reactive oxygen species (ROS) and activating the primordial follicle pool through inhibition of the PTEN pathway. This mechanistic difference explains why intrauterine infusion of PRP yields significant improvements in pregnancy rates, whereas intraovarian injection demonstrates greater potential in enhancing endocrine parameters (AMH/FSH).

### 5.2. Limitations of Current Mechanism Evidence

A notable limitation of the current mechanistic evidence is that the majority of signaling pathway data, including PI3K/Akt, TGF-β1-SMAD2/3, PTEN, and ROS scavenging pathways, are derived from preclinical models (rodent studies and in vitro experiments). While these pathways are biologically plausible and consistent with the known actions of platelet-derived growth factors, direct evidence in human endometrial and ovarian tissues remains limited. So, we believe future studies employing single-cell sequencing and spatial transcriptomics on human biopsy samples are needed to validate these mechanisms in the human reproductive tract.

### 5.3. Controversies over Standardization, Deficiency, and Dose Effects

The biggest challenge facing the current clinical translation of PRP is the lack of preparation and application standards. As shown in the literature review, there are huge differences among studies in terms of blood collection volume, centrifugation speed, activator use, and other aspects. This “black box” operation leads to high heterogeneity in platelet concentration multiples (usually 3–5 times [76]) and growth factor release profiles of PRP in different studies, severely impairing the comparability between studies.

Regarding the optimal dose, current evidence is contradictory. On the one hand, some studies suggest that 2 mL is a safe and effective dose, and excessive injection may cause ovarian tissue damage due to high pressure; on the other hand, data from patients with severe DOR show that a dose of ≥4 mL per ovary may be associated with better follicle recruitment effects [75]. This contradiction suggests that PRP may not have a simple linear dose–effect relationship but rather a “therapeutic window”. Future research urgently needs to establish an individualized dose calculation model based on ovarian tissue (assessed by 3D ultrasound) to avoid drug waste or tissue damage.

### 5.4. Safety Considerations and Future Prospects

Although PRP is derived from autologous blood and generally considered safe, its regenerative and mitogenic nature—characterized by high concentrations of VEGF, PDGF, IGF, and TGF-β—warrants a more comprehensive safety analysis beyond the current assertion that no serious adverse reactions have been observed. Severe infectious complications, including tubo-ovarian abscesses and Actinomyces abscess, have been reported following intraovarian PRP injection, although the true incidence remains unknown due to lack of systematic surveillance [77,78]. Beyond infection, intraovarian injection may also carry risks of ovarian tissue damage, bleeding, hematoma, and potential adhesion formation [79,80]. The mitogenic and angiogenic profile of PRP raises theoretical concerns about neoplastic cell proliferation, particularly in patients with hormone-dependent tumors: while no evidence currently links PRP to neoplastic transformation, and a 45-month follow-up of 89 breast cancer patients found no recurrence, the GRIIP 2025 formal consensus identifies certain types of oncologic diseases as a contraindication [80,81]. For patients at risk of hormone-dependent tumors, the long-term stimulatory effects of multiple angiogenic stimuli in PRP that promote tumor progression require longer follow-up data [82]. Given the lack of long-term safety data, PRP should be used with strict aseptic precautions and avoided in patients with active malignancy or hormone-dependent tumors. Future studies must systematically report adverse events, and large RCTs are urgently needed to confirm safety before routine clinical adoption.

Based on the above discussion, future research directions should focus on the following three points:

1. **Establishing a standardized system**: Promote multi-center collaboration to formulate relatively unified PRP preparations (such as using closed automatic separation systems) and quality control standards;
2. **Precision stratified treatment**: Clarify the optimal benefit thresholds for patients with different subtypes (such as different ages and different baseline FSH levels) through larger-scale RCTs;
3. **Exploration of combination therapies**: Combine single-cell sequencing technology to deeply analyze the specific action network of PRP in the reproductive microenvironment, and explore its synergistic effects with antioxidants, growth hormone, or stem cell therapies [83].

## 6. Conclusions

In conclusion, Platelet-Rich Plasma (PRP) has emerged as a promising regenerative modality in reproductive medicine, offering a novel therapeutic avenue for refractory infertility conditions. This review synthesizes evidence suggesting that PRP effectively addresses the core pathophysiological bases of thin endometrium and diminished ovarian reserve through multi-targeted mechanisms, including angiogenesis promotion, anti-inflammatory immunomodulation, and metabolic reprogramming. However, PRP’s clinical application remains investigational due to non-standardized preparation protocols, heterogeneous dosing and the predominantly preclinical nature of current mechanistic evidence. Establishing unified quality control standards and conducting large-scale multi-center randomized controlled trials are essential to define the therapeutic window, ensure long-term safety, and advance PRP from empirical black-box therapy to precision medicine in reproductive practice.

## Data Availability

No new data were created or analyzed in this study. Data sharing is not applicable to this article.

## Abbreviations

The following abbreviations are used in this manuscript:

ART	Assisted reproductive technology
TE	Thin endometrium
DOR	Diminished ovarian reserve
PRP	Platelet-rich plasma
IUA	Intrauterine adhesion
RIF	Recurrent implantation failure
CE	Chronic endometritis
POI	Premature ovarian insufficiency
VEGF	Vascular Endothelial Growth Factor
PDGF	Platelet-Derived Growth Factor
TGF-β	Transforming Growth Factor-beta
IGF	Insulin-like Growth Factor
IL	Interleukin
AFC	Antral follicle count
Ki-67	Antigen Ki-67
HOX	Homeobox
CCL	Chemokine (C-C Motif) Ligand
CXCL	Chemokine (C-X-C Motif) Ligand
COL1A1	Collagen Type I Alpha 1 Chain
TIMP	Tissue Inhibitor of Metalloproteinases
TNF-α	Tumor Necrosis Factor-alpha
MMP	Matrix Metalloproteinase
AMH	Anti-Müllerian Hormone
FSH	Follicle-Stimulating Hormone
ROS	Reactive Oxygen Species
PTEN	Phosphatase and Tensin Homolog
RCF	Relative Centrifugal Force
EDTA	Ethylenediaminetetraacetic acid
ACD	Acid-citrate Dextrose
GRIIP	International Research Group on Platelet Injections
